# Exercise metabolomics in pulmonary arterial hypertension: Where pulmonary vascular metabolism meets exercise physiology

**DOI:** 10.3389/fphys.2022.963881

**Published:** 2022-09-12

**Authors:** Michael H. Lee, Thaís C. F. Menezes, Julie A. Reisz, Eloara V. M. Ferreira, Brian B. Graham, Rudolf K. F. Oliveira

**Affiliations:** ^1^ Division of Pulmonary and Critical Care Medicine, Department of Medicine, University of California, San Francisco, San Francisco, CA, United States; ^2^ Division of Respiratory Diseases, Department of Medicine, Federal University of SP, São Paulo, Brazil; ^3^ Department of Biochemistry and Molecular Genetics, University of Colorado Anschutz Medical Campus, Aurora, CO, United States

**Keywords:** pulmonary hypertension, pulmonary arterial hypertension, exercise physiology, right heart catheterization, pulmonary vascular reserve, pulmonary vascular metabolism, metabolomics

## Abstract

Pulmonary arterial hypertension is an incurable disease marked by dysregulated metabolism, both at the cellular level in the pulmonary vasculature, and at the whole-body level characterized by impaired exercise oxygen consumption. Though both altered pulmonary vascular metabolism and abnormal exercise physiology are key markers of disease severity and pulmonary arterial remodeling, their precise interactions are relatively unknown. Herein we review normal pulmonary vascular physiology and the current understanding of pulmonary vascular cell metabolism and cardiopulmonary response to exercise in Pulmonary arterial hypertension. We additionally introduce a newly developed international collaborative effort aimed at quantifying exercise-induced changes in pulmonary vascular metabolism, which will inform about underlying pathophysiology and clinical management. We support our investigative approach by presenting preliminary data and discuss potential future applications of our research platform.

## Introduction

Pulmonary arterial hypertension (PAH), also known as group 1 pulmonary hypertension (PH) by the World Health Organization classification system, is a fatal condition characterized by aberrant proliferation of cells constituting the pulmonary vessels, resulting in vascular wall thickening and luminal obliteration to produce elevated pulmonary vascular resistance (PVR) and subsequent right ventricular (RV) failure (Stacher et al., 2012; Galie et al., 2015; Simonneau et al., 2019). Historically, PAH has been defined by hemodynamic indices measured with right heart catheterization (RHC) while the patient is at rest. PAH resting RHC diagnostic criteria include increased mean pulmonary artery pressure (mPAP) > 20 mmHg and elevated PVR (≥3 Wood units), along with pulmonary arterial wedge pressure (PAWP) ≤ 15 mmHg indicative of a normal left ventricular filling pressure.

Despite introduction of new therapies and improved survival, PAH remains incurable (Hurdman et al., 2012; McGoon et al., 2013; Hensley et al., 2018), and the anticipated 5-years survival of PAH patients can be as low as 50% (Hurdman et al., 2012). Notably, significant pulmonary vascular remodeling persists in the pulmonary arteries of PAH patients treated with modern PAH-targeted pharmacologic modalities (Stacher et al., 2012), underscoring shortcomings of current diagnostic and therapeutic approaches in detecting PAH at its initial stages and achieving reversal of pulmonary vascular remodeling. These rather disappointing outcomes suggest that 1) assessment of PAH based on resting hemodynamic evaluation may not properly characterize pulmonary vascular reserve and early pulmonary vascular dysfunction, and that 2) currently available treatments may not address all relevant pathogenetic mechanisms of PAH.

In this article, we explore two promising and interrelated areas of potential diagnostic and therapeutic implications in PAH: pulmonary vascular metabolism and pulmonary vascular physiology during exercise. We focus on recent discoveries and what they add to the current understanding of PAH pathophysiology and pathobiology. We further discuss an ongoing international collaboration to study pulmonary vascular metabolism during standardized exercise, and we report preliminary data highlighting the scientific and clinical relevance of our investigative work.

### Pulmonary vascular metabolism in healthy individuals and the O_2_ pathway

Daily life activities such as walking, talking and breathing depend on the maintenance of human body’s homeostasis. In turn, homeostasis depends on preserved tissue metabolism and the balance between energy production and consumption (Kisaka et al., 2017; Houstis et al., 2018). Human tissue metabolism is predominantly aerobic, using oxygen (O_2_) as the main substrate to regenerate adenosine triphosphate (ATP) from adenosine diphosphate (ADP), thereby generating energy for any given activity, even the involuntary ones, such as heart beating (Dhakal et al., 2015; Kisaka et al., 2017; Houstis et al., 2018).

All O_2_ utilized by the human body is absorbed at the pulmonary level and goes through different systems until tissue absorption; this process is called the O_2_ pathway (Dhakal et al., 2015; Houstis et al., 2018). The O_2_ pathway involves alveolar ventilation, diffusion into pulmonary capillary blood and hemoglobin, blood flow through the systemic circulation, diffusion into the tissue cells and, finally, mitochondrial respiration (Dhakal et al., 2015; Houstis et al., 2018). Therefore, the interactions among cardiac, pulmonary vascular, hematologic, and nervous systems are essential to human body metabolism and energy production (Dhakal et al., 2015; Huang et al., 2017; Kisaka et al., 2017; Melamed et al., 2019; Cho et al., 2020; Grafton et al., 2020; Malte et al., 2021). The O_2_ carried via the O_2_ pathway and absorbed by the cells can be represented and quantified as O_2_ uptake (VO_2_). According to the Fick principle, VO_2_ is the product of cardiac output (CO) and the arterial-mixed venous O_2_ content difference (C_(a-v)_O_2_) (Dhakal et al., 2015; Kisaka et al., 2017; Melamed et al., 2019). Arterial O_2_ content (CaO_2_), can be calculated by: 1.36 x Hb x SaO2+0.003 x PaO2 , in which SaO_2_ and PaO_2_ represent arterial O_2_ saturation and pressure, respectively (Melamed et al., 2019). VO_2_ directly influences one’s aerobic capacity and their ability to tolerate physical exercise.

Upstream of cellular metabolism, O_2_ must be adequately absorbed from the external environment and transported to all other systems (Tabima et al., 2017; Houstis et al., 2018). In this context, the pulmonary and cardiovascular systems work as a unit and play an important and interdependent role in the O_2_ pathway (Tabima et al., 2017). At the lung level, the absorption of O_2_ occurs through the alveolar-capillary membrane; adequate function of this membrane depends on membrane conductance (directly related to pulmonary vascular wall integrity), pulmonary capillary blood volume (directly related to pulmonary vascular blood flow, and therefore RV function), and the degree of remodeling and thickness of the alveolar capillaries (Olson et al., 2016). From a physiological standpoint, it is essential that the alveolar, pulmonary vascular, and cardiac systems work properly and that the interactions among these components are preserved for adequate function of the O_2_ pathway (Tabima et al., 2017).

Given that pulmonary vascular membrane conductance is highly dependent on the integrity of pulmonary vascular wall tissue, conditions that stimulate excessive growth of pulmonary vascular cells, such as PAH, will affect the diffusion capacity of the alveolo-capillary membrane. As a result, via pulmonary vascular remodeling and associated pulmonary vascular stiffness (Singh et al., 2019a), PAH leads to progressive obliteration of pulmonary arterial lumen, subsequently raising PVR and therefore increasing PAP, ultimately resulting in a dysfunctional RV (Naeije and Chesler, 2012; Naeije, 2013; Dhakal et al., 2015; Olson et al., 2016; Kisaka et al., 2017; Tabima et al., 2017), which in sum adversely impacts the O_2_ pathway.

### Pulmonary vascular metabolism in pulmonary arterial hypertension at the cellular level

In a normal, disease-free state, metabolism plays an important role in maintaining the integrity of vascular structure and function. The pulmonary vasculature is comprised of distinct cell types working in concert, including endothelial cells, smooth muscle cells, and perivascular stromal and immune cells. In each of these cell types, a number of metabolic pathways involving various metabolites, such as glucose, glutamine, and fatty acids among others, regulate cellular functions. For example, in endothelial cells, glycolysis is crucial in the preservation of adherens junctions, repair after injury, and new vessel formation, in addition to ATP generation (Culic et al., 1997; De Bock et al., 2013; Li et al., 2019; Stevens et al., 2021). Interplays among metabolic pathways is similarly important in the homeostasis of the vascular smooth muscle cells (Stenmark et al., 2018) and surrounding immune cells (O'Neill et al., 2016). Interactions between different cell types is also crucial for physiologic function of the pulmonary vasculature (Gao et al., 2016), such as the production of nitric oxide by endothelial cells which regulates smooth muscle cell tone.

A sizeable body of literature on PAH in humans and PH in animal models have underscored an important role of dysregulated and altered pulmonary vascular cell metabolism in disease pathogenesis (Tuder et al., 2012; Cottrill and Chan, 2013; Sutendra and Michelakis, 2014). Historically, the role of dysregulated metabolism as a key driver of disease was first established in cancer studies before its relevance was recognized in pulmonary vascular diseases. In fact, metabolic dysregulation represents one of several pathobiologic similarities between cancer and PAH, comprising a paradigm known as the cancer hypothesis of PAH (Lee et al., 1998; Voelkel et al., 1998; Rai et al., 2008). This hypothesis originated from the observation that proliferating endothelial cells in pulmonary arterial plexiform lesions (a characteristic pulmonary vascular pathology) of idiopathic PAH (IPAH) patients are clonal, suggesting cancer-like selection for cells with genetic growth advantages (Lee et al., 1998); it was subsequently expanded by the recognition of somatic mutations and growth-promoting gene expressions in PAH lungs.

Notably, many pro-survival and pro-proliferation signals affect cell metabolism, and conversely, metabolic dysregulations can have growth-inducing effects. Although once believed to represent passive consequences of cancer-promoting signals (Levine and Puzio-Kuter, 2010), it is now understood that modulation of metabolic pathways can epigenetically program cell behavior, thereby actively influencing tumor growth (Wellen and Thompson, 2012; Hirschey et al., 2015). Given the overlaps between PAH and cancer, and considering that PAH is a disease marked by intimal cell proliferation and medial hypertrophy (Stacher et al., 2012), it is likely that altered pulmonary vascular metabolism significantly contributes to PAH pathobiology.

One well known example of dysregulated cellular metabolism affecting both cancer and PAH is the shift of glucose metabolism toward glycolysis accompanied by reduced mitochondrial glucose oxidation, a phenomenon termed aerobic glycolysis or the Warburg effect (Koppenol et al., 2011). Glucose uptake and glycolysis are increased in the lungs of PAH patients, both at the whole lung (Xu et al., 2007; Hagan et al., 2011; Zhao et al., 2013) and the cellular levels (Fijalkowska et al., 2010; Hernandez-Saavedra et al., 2020). In animal models of experimental PH, both pharmacologic global inhibition of glycolysis (Michelakis et al., 2002; McMurtry et al., 2004; Guignabert et al., 2009) and cell-specific deletion of pro-glycolytic genes (Ball et al., 2014; Cao et al., 2019; Kojima et al., 2019; Luo et al., 2019; Wang et al., 2021) were protective. One mechanism by which cytoplasmic glucose oxidation supports cell growth and proliferation is a resulting flux of glucose carbons into the pentose phosphate pathway, including NADPH production, antioxidant glutathione regeneration, and lipid membrane and nucleotide synthesis (Vander Heiden et al., 2009; Weinberg et al., 2010; Lunt and Vander Heiden, 2011; De Bock et al., 2013). The pathobiological relevance of other glycolysis-related processes, such as lactate fermentation, in PAH will require further studies.

Another group of metabolites that likely contributes to PAH pathobiology is fatty acids. Studies suggest fatty acid metabolism and lipotoxicity-induced promotion of PAH is multi-dimensioned. For instance, fatty acid oxidation is increased at the whole body level in PAH patients (Mey et al., 2020), but it appears to be reduced specifically in their RVs (Hemnes et al., 2014; Brittain et al., 2016; Legchenko et al., 2018); RV fatty acid uptake might be disease-specific (Graham et al., 2015; Talati et al., 2016). Notably, strategies to induce (Legchenko et al., 2018) and inhibit (Sutendra et al., 2010; Lee et al., 2022) fatty acid oxidation at the systemic level both attenuated experimental PH in animals. Collectively, these conflicting observations indicate the complexity underlying the mechanistic link between fatty acid metabolism and PAH, and underscore the importance of probing disease and tissue-specific (e.g., PA vs. RV) roles of fatty acid metabolism. In addition to glucose and fatty acids, other metabolic pathways are also critical in PH pathobiology, including amino acid metabolism and the Krebs cycle, among others (Xu et al., 2021).

The long-recognized link between metabolism and cell growth has been targeted in the cancer field, in which metabolism-altering treatments have been heavily investigated in clinical trials with some approved for clinical use (Luengo et al., 2017; Stine et al., 2022). Despite a large body of evidence describing the pathogenetic role of metabolism in the pulmonary vasculature and the clinical availability of many metabolism-modulating agents, clinical applications targeting metabolic pathways in PH have been limited to a handful of studies (Bhandari and Subramanian, 2007; Finch et al., 2016; Michelakis et al., 2017). For example, by shifting glucose metabolism from glycolysis into the Krebs cycle, the pharmacologic compound dichloroacetate produced clinical improvement in PAH, but this beneficial effect was particularly observed in a subset of susceptible human subjects who were shown not to carry specific pro-glycolytic mutations (Michelakis et al., 2017).

One plausible explanation of this hindrance to clinically targeting metabolism in PAH is our currently limited understanding of how pulmonary vascular metabolism changes with exercise in health and disease, and how it relates to physiology particularly as blood flow through the pulmonary circulation, CO, and VO_2_ simultaneously increase during physical exertion. Given that dysregulated metabolism is reflective of pulmonary vascular remodeling, which in turn dictates pulmonary vascular and RV reserves, evaluating pulmonary vascular metabolism during exercise is anticipated to provide important advantages. For example, stressing the cardiopulmonary system toward the limit of its reserve may reveal underlying metabolic dysregulations which are not apparent at rest to a now quantifiable level, improving the sensitivity performance of the measurements. Exercise allows a dynamic assessment of the pulmonary vascular reserve in addition to metabolic pathways. Lastly, and perhaps most importantly, studying pulmonary vascular metabolism during exercise allows the identification of pertinent metabolic targets that correlate with exercise capacity, a well-stablished marker of disease severity (Galie et al., 2015), which may point to new pathobiologic targets for further investigation. Before exploring how the interplay between pulmonary vascular physiology and metabolism can be quantified in PAH patients during exercise, we first discuss the two components individually.

### Pulmonary vascular physiology in pulmonary arterial hypertension during exercise

Under physiologic conditions, the pulmonary vasculature and the RV are coupled (Singh et al., 2019b; Singh et al., 2020; Singh et al., 2021). RV function depends on its ability to contract (muscular preserved contraction), its preload, and its afterload (i.e., the pulmonary vasculature) (Naeije, 2013; Tabima et al., 2017). The afterload imposed on the RV by the pulmonary vasculature is comprised of several components, including vascular resistance to blood flow, vascular compliance, pulsatile blood flow (i.e., arterial wave reflections) and blood inertance (Tedford, 2014). All these components are influenced by passive (transmural pressure driven by input and outflow pressures, i.e., PAP and PAWP, respectively) and active (sympathetic nervous system stimulation, nitric oxide-mediated mechanisms, and regional alveolar hypoxia) factors at rest, which are exacerbated during exercise (Reeves et al., 1988). Passive factors assist in ventilation and perfusion matching, while active factors dynamically regulate exercise vasodilation and vasoconstriction, further influencing pulmonary vascular distension (Kadowitz and Hyman, 1973; Kane et al., 1994; Reeves et al., 2005). Under physiologic conditions, the pulmonary vasculature is a system of low resistance and high compliance, with the ability to stretch and accommodate with minimal resistance increasing blood flow as CO dynamically rises (Naeije, 2013; Tabima et al., 2017). Therefore, while at rest, the RV requires minimal energy expenditure to eject blood flow through the normal pulmonary vasculature.

In healthy individuals, exercise-induced increases in heart rate and stroke volume result in augmentation of CO (Kovacs et al., 2009; Naeije and Chesler, 2012; Naeije, 2013; Tabima et al., 2017). The increased pulmonary vascular blood flow due to the increased CO raises PAP, left atrial pressure, and right atrial pressure. The increase in left atrial pressure during exercise influences PAP dynamic changes and its pulsatile component (Naeije, 2013; Olson et al., 2016; Tabima et al., 2017). The balance among these forces during exercise is preserved in healthy individuals (Naeije, 2013). However, the pulmonary vascular response during exercise may change with aging (Kovacs et al., 2009; Naeije, 2013; Oliveira et al., 2016a). As a result of the exercise-related increased pulmonary vascular blood flow, PVR will decrease during exercise in heathy individuals, mainly due to dilation of pulmonary vessels and recruitment of unperfused ones (Kovacs et al., 2012; Langleben et al., 2018; Olschewski et al., 2018). As the vessels distend due to the increased pulmonary vascular blood flow, their capacity to further accommodate the increasing blood flow also decreases, resulting in reduction of pulmonary vascular compliance (PVC) (Kovacs et al., 2009; Kovacs et al., 2012; O'Neill and Johnson, 1991; Oliveira et al., 2020). Similar to mPAP/CO exercise responses, older subjects tend to have higher PVR and lower PVC compared with younger individuals (Naeije, 2013; Oliveira et al., 2016a). The higher PVR and lower PVC in the elderly may be explained in part by the greater vascular stiffness associated with aging, and the more pronounced exercise-induced sympathetic nervous system stimulation during physical effort (Naeije, 2013; Oliveira et al., 2016a).

The development of abnormal pulmonary vascular responses to exercise is associated with a reduced exercise capacity. In PAH, exercise intolerance is specifically associated with impaired O_2_ delivery (DO_2_) and indices of increased RV and pulmonary vascular load (Oliveira et al., 2017). A decreased DO_2_ is in turn associated with concurrent reductions of exercise CaO_2_ and exercise CO. The reduction of CaO_2_ levels likely reflects the known effect of pulmonary vascular diseases on O_2_ diffusion in the pulmonary vasculature (Sun et al., 2003). Along these lines, a decreased RV stroke work index (RVWSI) at peak exercise concurrent with a dynamically reduced pulmonary vascular reserve evident by an impaired exercise PVC is associated with decreased peak VO_2_ (Messina et al., 2021). Failure to appropriately reduce PVR in relation to PVC during exercise due to pulmonary vascular remodeling has been shown to be a potential exercise hallmark of PH (Oliveira et al., 2020). As an early marker of pulmonary vascular remodeling, a dysfunctional PVC during exercise suggests that changes in intrinsic elastic properties of the pulmonary circulation, in addition to increased transmural pressure, underlie the increase in pulmonary vascular stiffness and its association with PH severity (Sanz et al., 2009). Therefore, by analyzing hemodynamic patterns under the stress of exercise, it is possible to infer pulmonary vascular reserve in PAH patients. How exercise-induced hemodynamic changes precisely relates to pulmonary vascular metabolism in PAH, however, remains uncharacterized.

### Pulmonary vascular metabolism in pulmonary arterial hypertension during exercise

Most pulmonary vascular metabolic studies to date have focused on metabolism at rest, whereas PAH patients experience symptoms with exertion. In this context, metabolic phenotypes of the pulmonary vasculature in PAH may also become more apparent during exercise, particularly given that exercise capacity in PAH is a direct reflection of pulmonary vascular and RV reserve (Kovacs et al., 2017). The clinical relevance of assessing and correlating hemodynamic changes and pulmonary vascular metabolism during exercise is underscored by studies of patients with heart failure with preserved ejection fraction, in which effects of the nitric oxide donor, sodium nitrite, became much more apparent during exercise compared to at rest (Borlaug et al., 2015).

**FIGURE 1:**
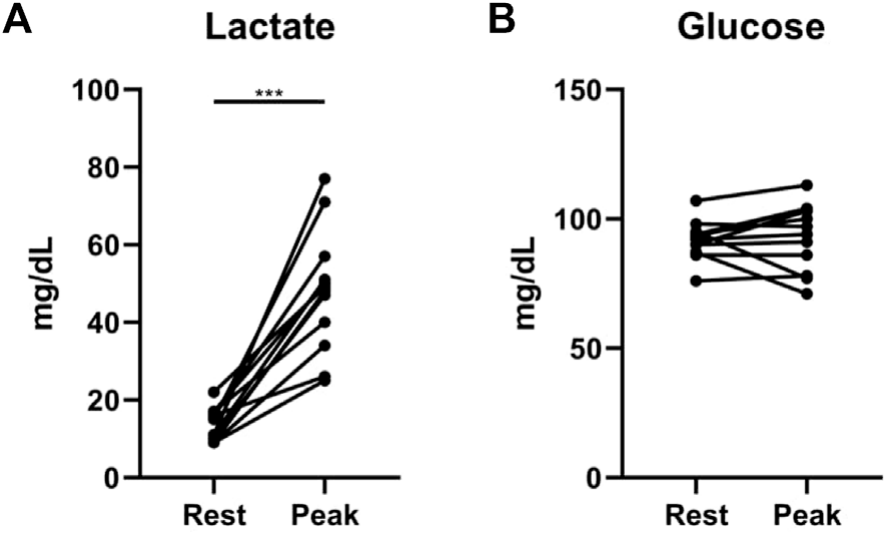
Exercise-induced changes in lactate **(A)** and glucose **(B)** levels from rest to peak exercise, measured from pulmonary artery catheters in 12 patients with systemic sclerosis-associated pulmonary arterial hypertension. ***p = 0.0005 (Wilcoxon matched-pairs signed rank test).

**FIGURE 2:**
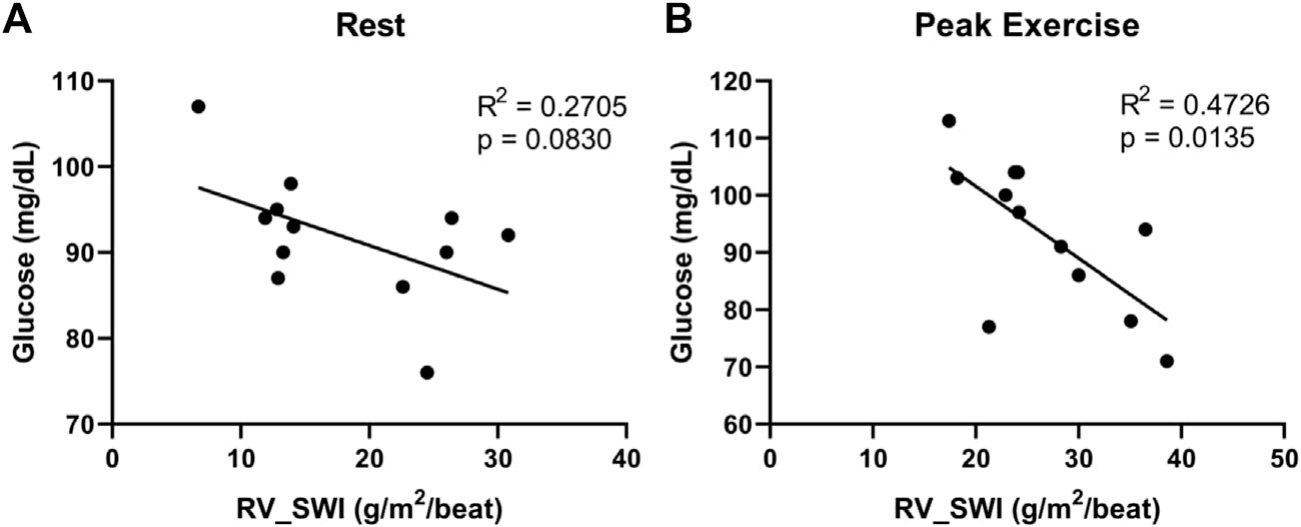
Correlations between right ventricular stroke work index (RV_SWI) and pulmonary artery glucose levels, measured at rest **(A)** and peak exercise **(B)** in 12 patients with systemic sclerosis-associated pulmonary arterial hypertension.

We found additional evidence supporting the importance of interrogating exercise-induced metabolic changes. In a cohort of 12 non-diabetic patients with systemic sclerosis-associated PAH (SScPAH) who underwent clinically indicated exercise RHC, we measured PA glucose and lactate levels both at rest and at peak exercise, along with routine hemodynamic indices. As anticipated, PA lactate levels increased with exercise, but PA glucose levels did not change (Figure 1). Interestingly, we found at peak exercise an inverse correlation between PA glucose and RVSWI (Figure 2), a load-dependent measurement of RV contractility that reflects the amount of work (i.e., energy expenditure) required by the RV to generate its stroke volume while overcoming the PA pulsatility and hemodynamic oscillations (Brittain et al., 2013; Messina et al., 2021). The data indicate a potential pathobiological relationship between PA glucose metabolism and RV function during exercise. This observation is consistent with the current knowledge that RV glucose uptake is increased in PAH patients, as demonstrated by imaging studies (Oikawa et al., 2005). However, it is unknown if this finding is a reflection of RV metabolic shift, increased stroke work or RV ischemia (Sanz et al., 2019), or a combination of all these factors.

### The potential of transpulmonary metabolomics in pulmonary arterial hypertension during exercise

While previous studies of metabolic changes in PAH patients, both at the cellular (Fessel et al., 2012) and the systemic levels (Lewis et al., 2016; Rhodes et al., 2017), have yielded valuable information, clinical application of these findings have been limited, largely due to the lack of metabolic information specific to the pulmonary vasculature and during exercise when patients are most symptomatic and hemodynamic changes are most pronounced, as discussed above. We have thus developed an international collaborative effort which aims to study transpulmonary metabolite flux during standardized exercise.

One guiding principle of our method is that differences between paired blood draws across a tissue bed can quantify net metabolite uptake versus excretion from that bed. Specifically, reflecting the direction of blood flow, subtracting the concentration of each metabolite measured in blood drawn from a PA catheter (pulmonary circulation) from that in blood drawn from a radial arterial line (systemic circulation) allows the quantification of net metabolic flux (i.e., excretion or uptake) occurring across the pulmonary vascular bed. In this manner, simultaneously collecting blood samples from the PA catheter and the radial arterial line enables specific quantification of pulmonary vascular metabolic flux (Lewis et al., 2016).

Another novel aspect of our experimental approach is the assessment of pulmonary vascular metabolism at distinct, clinically relevant stages of standardized exercise, for example during freewheeling (load-free) and post-exercise recovery, in addition to at rest and peak exercise. Exercise endpoints measured during freewheeling were shown to have prognostic value in PAH (Sayegh et al., 2020), and delayed recovery rates are anticipated in more severe PAH (Ramos et al., 2012; Oliveira et al., 2016b). We therefore hypothesize that metabolomic interrogation during freewheeling and in recovery, in addition to the classical at rest (pre-exercise) and peak exercise timepoints, will demonstrate pathobiological and prognostic significance of changes in pulmonary vascular metabolism in response to changing exercise load.

**FIGURE 3:**
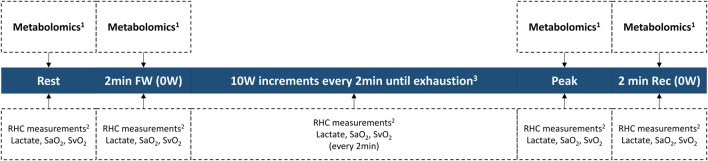
Invasive exercise protocol. ^1^Collected from the pulmonary artery catheter and from the radial artery catheter. ^2^RHC measurements include heart rate, blood pressure, right atrial pressure, pulmonary arterial pressure, pulmonary arterial wedge pressure, and cardiac output. ^3^Study length varies on an individual basis, until exhaustion. *RHC right heart catheterization; SaO2 radial artery O2 saturation; SvO2 mixed-venous O2 saturation; workload in Watts*.

Our investigative effort represents a teamwork of clinician-scientists and basic scientists in Brazil and the United States. We plan to prospectively enroll PAH patients who will undergo clinically indicated exercise RHC at the Invasive Pulmonary Hemodynamic Assessment Program at the Federal University of São Paulo in Brazil (Ramos et al., 2012; Oliveira et al., 2014a; Oliveira et al., 2014b; Ramos et al., 2014; Oliveira et al., 2018; Vieira et al., 2020; Messina et al., 2021). Briefly, a resting supine RHC will be first performed following standard recommendations (Kovacs et al., 2013; Galie et al., 2015; Kovacs et al., 2017). Next, patients will exercise on a supine cycle ergometer attached to the table, following an incremental step-test with a 10 W increase every 2 min until exhaustion. Measurements include radial artery, right atrial, PA, and PA wedge pressures, as well as radial and pulmonary artery O_2_ saturations (Boerrigter et al., 2014; Herve et al., 2015; Oliveira et al., 2016a; Oliveira et al., 2016b; Kovacs et al., 2017; Oliveira et al., 2017). CO will be measured in triplicate at rest and once per exercise stage using thermodilution. Our exercise protocol is summarized in Figure 3.

During the study, paired blood samples will be simultaneously collected from the PA catheter and the radial arterial line at the four specified timepoints of clinical relevance (at rest, after 2 min of freewheeling, at peak exercise, and 2 min into recovery). Blood samples will be centrifuged at +4 °C, and the plasma aliquoted in preparation for a mass spectrometry-based metabolomic assessment. Subsequently, the plasma samples will be analyzed and profiled for changes in metabolites central to energy metabolism by the Metabolomics Core at the University of Colorado (United States) (Graham et al., 2018; Hernandez-Saavedra et al., 2020). Untargeted data will be mined in conjunction with the KEGG database for metabolites in carbon and nitrogen metabolism (identifying ∼175 metabolites) (Nemkov et al., 2017; Nemkov et al., 2019). This platform has been successfully applied to multiple studies on human plasma specimens (Thomas et al., 2020; Ye et al., 2021). The transpulmonary gradient of each metabolite is determined by subtracting the value measured in the PA from that in the radial artery, reflecting net uptake or excretion by the pulmonary vascular bed.

Exercise-induced changes in transpulmonary metabolite gradients incorporating both the systemic and the pulmonary circulations have not been reported to date, underscoring the novelty of our experimental design. Moreover, significant metabolomic findings from pulmonary arterial blood samples alone, reported by our group (Figures 1, 2) and others (Sanders et al., 2019), add confidence that our approach will yield invaluable information clarifying the contribution of pulmonary vascular metabolism to PAH pathobiology. It is worth mentioning that similar efforts at quantifying the pulmonary vascular metabolome during exercise are being undertaken by other groups, including the PVDOMICS initiative in the United States (Hemnes et al., 2017; Tang et al., 2020). Given that our patient population consists of Brazilian citizens in São Paulo, who are ethnically and therefore genetically extremely diverse, we expect to make discoveries that are both unique and complementary to those from other patient cohorts. Additionally, our experimental design will capture, for the first time, low-intensity, resistance-free exercise (freewheeling), thereby providing new insights into how routine, non-strenuous daily activities might relate to pulmonary vascular metabolism in PAH patients.

In summary, we propose to perform high-throughput mass spectrometry-based metabolomics on blood samples obtained at four pre-specified stages of standardized exercise. We anticipate that simultaneous, high-throughput measurement of metabolites excreted or taken up by the pulmonary vascular bed will define relationships among pulmonary vascular metabolism, hemodynamic indices of PAH severity, and the degree of underlying pulmonary vascular remodeling and functional reserve. Our approach will allow the correlation of metabolomics with clinical endpoints of pulmonary vascular and exercise physiology, potentially serving as a novel therapeutic and diagnostic tool in managing PAH patients.

## Conclusion

The pathophysiology of PAH represents a complex interplay among cardiopulmonary physiology, the O_2_ pathway, and pulmonary vascular metabolism at the cellular level. Disturbances to each of these individual factors collectively reflect the underlying pathobiology of remodeled pulmonary vasculature, and exercise creates a unique environment allowing a dynamic assessment of pulmonary vascular metabolism and identification of relevant metabolic processes. Interrogating pulmonary vascular metabolism in the context of exercise capacity and hemodynamic variables will clarify how metabolism contributes to PAH and help establish novel diagnostic strategies and rational treatment targets based on both molecular and physiologic parameters.

To investigate these topics, we propose a study that has the potential to significantly impact our understanding of PAH vascular metabolism and the care of PAH patients. Establishment of clinically and pathobiologically important metabolic targets that closely correlate with both physiologic and pathologic metrics will serve as a prognostic tool to monitor disease progression and treatment response. If this platform is effective, metabolism-modulating therapies might be tested for effectiveness in the setting of standardize exercise. Additionally, metabolic characterization may prove useful in selecting at-risk patients who may benefit from close follow-up or early initiation of treatment. Although our focus at this time is on PAH, the same exercise protocol and method of analysis can be used to provide useful insights into disease pathophysiology in other PH etiologies in the future. We plan to bring together the two areas of established relevance in PAH, dysregulated metabolism and exercise physiology, and interrogate their intersection.
